# Supplementary material for: Caspase-8 contributes to angiogenesis and chemotherapy resistance in glioblastoma
Source: eLife. 2017 Jun 8;6:e22593. doi: 10.7554/eLife.22593 (PMC5464770; doi:10.7554/eLife.22593)
Supplement: Figure 2—source data 4. — The Pearson correlation coefficients and corresponding p-values between Caspase-8 expression and those of the different cytokine and growth factor genes are displayed. DOI: http://dx.doi.org/10.7554/eLife.22593.014 [file elife-22593-fig2-data4.pdf]

Cytokines	Pearson Correlation	P-Value
MCP-1	0.527	7.900E-15
IL-1β	0.309	3.400E-05
IL-6	0.252	7.900E04
IL-8	0.269	3.300E-04
VEGF	0.093	2.240E-01

Figure 2_source data 4
