# Supplementary material for: Caspase-8 contributes to angiogenesis and chemotherapy resistance in glioblastoma
Source: eLife. 2017 Jun 8;6:e22593. doi: 10.7554/eLife.22593 (PMC5464770; doi:10.7554/eLife.22593)
Supplement: Supplementary file 1. — DOI: http://dx.doi.org/10.7554/eLife.22593.027 [file elife-22593-supp1.docx]

**Supplementary Materials and Methods**

**The Cancer Genome Atlas data retrieval**

The Cancer Genome Atlas (TCGA) data are included in the NCI Genomics Data Common (GDC, gdc.cancer.gov) and can be accessed through the GDC Legacy Archive Portal (https://portal.gdc.cancer.gov/legacy-archive/search/f). To retrieve the data used in this work, the user can combine a number of search terms with the Boolean operator AND, as follows:

*Disease Type IS Glioblastoma Multiforme AND Primary Site IS Brain AND Program Name IS TCGA AND Project Id IS TCGA-GBM AND Access IS open AND Data Category IS Gene expression AND Data Format IS TXT AND Data Type IS Gene expression quantification AND Experimental Strategy IS RNA-Seq AND Platform IS Illumina HiSeq*

When the user sets these search parameters, a list of files matching the query is provided, and individual files can be added to a download cart. Then, a manifest file is created that contains information needed by a download software tool, provided by the GDC itself, that would actually download the selected data.

Moreover, a URL is created by the query that allows direct access to the same files. The URL generated by our setting is:

<https://portal.gdc.cancer.gov/legacy-archive/search/f?filters=%7B%22op%22:%22and%22,%22content%22:%5B%7B%22op%22:%22in%22,%22content%22:%7B%22field%22:%22cases.project.program.name%22,%22value%22:%5B%22TCGA%22%5D%7D%7D,%7B%22op%22:%22in%22,%22content%22:%7B%22field%22:%22cases.project.primary_site%22,%22value%22:%5B%22Brain%22%5D%7D%7D,%7B%22op%22:%22in%22,%22content%22:%7B%22field%22:%22cases.project.project_id%22,%22value%22:%5B%22TCGA-GBM%22%5D%7D%7D,%7B%22op%22:%22in%22,%22content%22:%7B%22field%22:%22cases.project.disease_type%22,%22value%22:%5B%22Glioblastoma%20Multiforme%22%5D%7D%7D,%7B%22op%22:%22in%22,%22content%22:%7B%22field%22:%22files.data_category%22,%22value%22:%5B%22Gene%20expression%22%5D%7D%7D,%7B%22op%22:%22in%22,%22content%22:%7B%22field%22:%22files.data_type%22,%22value%22:%5B%22Gene%20expression%20quantification%22%5D%7D%7D,%7B%22op%22:%22in%22,%22content%22:%7B%22field%22:%22files.experimental_strategy%22,%22value%22:%5B%22RNA-Seq%22%5D%7D%7D,%7B%22op%22:%22in%22,%22content%22:%7B%22field%22:%22files.platform%22,%22value%22:%5B%22Illumina%20HiSeq%22%5D%7D%7D%5D%7D>

The query retrieves 348 files. In particular, among the files retrieved by this query, we employed the files reporting gene-level RSEM normalized expression, which can be recognized since the file name ends with rsem.genes.normalized_results.
